# Effects of consumption of coffee, tea, or soft drinks on open-angle glaucoma: Korea National Health and Nutrition Examination Survey 2010 to 2011

**DOI:** 10.1371/journal.pone.0236152

**Published:** 2020-07-20

**Authors:** Jeong Hun Bae, Joon Mo Kim, Jung Min Lee, Ji Eun Song, Mi Yeon Lee, Pil-Wook Chung, Ki Ho Park

**Affiliations:** 1 Department of Ophthalmology, Kangbuk Samsung Hospital, Sungkyunkwan University School of Medicine, Seoul, Republic of Korea; 2 Division of Biostatistics, Department of Medical Information, Kangbuk Samsung Hospital, Seoul, Republic of Korea; 3 Department of Neurology, Kangbuk Samsung Hospital, Sungkyunkwan University School of Medicine, Seoul, Republic of Korea; 4 Department of Ophthalmology, Seoul National University College of Medicine, Seoul, Republic of Korea; University of California San Diego, UNITED STATES

## Abstract

We sought to investigate the association between consumption of coffee, tea, or soft drinks and risk of open-angle glaucoma (OAG) among Koreans using nationwide population-based data. This cross-sectional survey was performed through the Korea National Health and Nutrition Examination Survey 2010 to 2011. Participants older than 19 years were included in the sample for analysis after excluding those with any missing data. The diagnosis of OAG was based on the International Society of Geographical and Epidemiological Ophthalmology criteria, and participants without glaucomatous optic neuropathy served as controls. The frequency of beverage consumption during the past 12 months was obtained through a questionnaire. Multivariate logistic regression models were used to determine the relationship between consumption of each type of beverage and prevalence of OAG. A total of 6,681 participants was included in the analysis. The prevalence of OAG was 4.4% (n = 323), including 5.4% (n = 169) among men and 3.5% (n = 154) among women. After adjusting for multiple covariates, coffee consumption was significantly associated with OAG, while no significant association was found between consumption of tea or soft drinks and OAG. Participants who drank coffee had a higher risk of having OAG compared with those who did not drink coffee (odds ratio [OR], 2.40; 95% confidence interval [CI], 1.22–4.72; p = 0.011). In sex-stratified analyses, the robust association of coffee consumption with OAG was observed in men (OR, 3.98; 95% CI, 1.71–9.25; p = 0.001) but not in women. Our results suggest that coffee consumption may affect the risk of OAG, particularly in men.

## Introduction

Relatively high intraocular pressure (IOP) can have a negative effect on the optic nerve and is the most important cause of development and progression of glaucoma. Therefore, most studies suggest that lowering IOP in glaucoma patients can prevent glaucoma progression. However, it is also true that glaucoma development or progression can occur even if the IOP is within the normal range. Many studies have been conducted to identify other solutions for patients who cannot be managed by lowering IOP [1,2]. In addition to this, various other risk factors affecting glaucoma have been reported [3–6].

Although the effects of environmental factors on the development or progression of glaucoma may be evident, the associations are weak, with no clear evidence [3–6]. Despite the demonstrated importance of low IOP in glaucoma, patients often seek other methods that offer favorable effects on glaucoma. Many people wonder if it is possible to stabilize the glaucoma state by changing daily lifestyle in addition to pursuing IOP control.

Caffeine is a widely consumed ingredient worldwide, and studies have reported equivocal effects on glaucoma [7–11]. Some research has indicated that caffeinated coffee consumption increases the risk of glaucoma associated with elevated IOP and plasma homocysteine level [11–14]. Studies about the association of commonly consumed beverages with glaucoma demonstrated equivocal associations, and the effect could be different among ethnicities or individuals. Thus, in this study, we sought to investigate the association of consumption of coffee, tea, and soft drinks typical in Korea with open-angle glaucoma (OAG) using the data from a nationwide population-based survey.

## Materials and methods

### Data source and study participants

This study was based on data from the Korea National Health and Nutrition Examination Survey (KNHANES) 2010 to 2011, which is an ongoing, nationwide population-based survey conducted periodically by the Korea Centers for Disease Control and Prevention (KCDCP) and the Korean Ministry of Health and Welfare. The data from the KNHANES are nationally representative of noninstitutionalized civilians in Korea. Participants were randomly selected through a stratified, multistage, probability-sampling design according to sampling units based on age group from household registries and economic status, sex, and geographical area. The study design of the KNHANES has been described in detail elsewhere [15]. All participants provided written informed consent to participate in the study, and the KNHANES studies were conducted according to the guidelines put forth in the Declaration of Helsinki. The study protocol was approved by the Institutional Review Board of the KCDCP. As the KNHANES data are deidentified and publicly available on the KNHANES website (http://knhanes.cdc.go.kr), this study was exempt from required approval by the Institutional Review Board of Kangbuk Samsung Hospital.

A total of 17,476 participants was enrolled in the KNHANES 2010 to 2011. Of these, we excluded participants if they were younger than 19 years; pseudophakic or aphakic; and/or had a history of retinal or refractive surgery, evidence of retinal detachment, signs of macular degeneration or diabetic retinopathy on examination, or a history of cerebrovascular disease that may affect visual field results. Participants with OAG treated with anti-glaucoma medication or surgery, with other types of glaucoma than OAG, or with any missing data were also excluded. Finally, a total of 6,681 participants was included in the analysis.

### Data collection and definitions of variables

The KNHANES had three component surveys: a health interview, a health examination, and a nutrition survey. The survey response rate was 76.1% for the health interview and examination survey and 82.4% for the nutrition survey [15]. Information on demographics, health behaviours (physical activity, smoking, and alcohol consumption), and medical conditions (history of physician-diagnosed disease, current medications) was collected during the health interview. Health behaviours were assessed using questions about habits during a one-month period before the interview. After the interview, height and body weight were measured with the participants wearing light clothing and no shoes. Body mass index (BMI) was calculated as weight in kilograms divided by square of height in meters. Waist circumference was measured at the narrowest point between the lower border of the rib cage and the iliac crest.

Physical activity classification was based on the International Physical Activity Questionnaire short-form scoring protocol, and a participant’s physical activity was classified as ‘regular physical activity’ when they were engaged in moderate-intensity activity more than five times per week or in vigorous activity more than three times per week [16]. Smoking status was classified as ‘current smoker’ (more than 100 cigarettes over the lifetime and current smoking status) or ‘non-smoker’, while alcohol consumption was categorized as ‘heavy drinking’ at more than 60 g/day in men or more than 40 g/day in women more than two days per week or ‘other’.

In the KNHANES, participants were asked to respond to questions about the frequency of beverage consumption during the past 12 months. For consumption of coffee, the survey question was “How often did you drink a cup of coffee?”. Response options included none, six to 11 cups per year, one cup per month, two to three cups per month, one cup per week, two to three cups per week, four to six cups per week, one cup per day, two cups per day, and three or more cups per day. The same question was asked about tea and soft drinks, with the same frequency options. The questionnaire did not classify caffeinated or non-caffeinated beverages.

### Ophthalmological examination

All participants underwent detailed ocular examinations, including measurement of visual acuity and IOP, autorefraction, slit-lamp biomicroscopy, and fundus photography. Certified ophthalmologists performed all ocular examinations, and the Epidemiologic Survey Committee of the Korean Ophthalmologic Society verified the quality of the ophthalmic surveys [17]. Slit-lamp biomicroscopy was performed for detection of anterior segment pathologies and assessment of peripheral anterior chamber depth (PACD) using the Van-Herick method. Fundus photographs were produced with a digital non-mydriatic fundus camera (TRC-NW6S; Topcon, Tokyo, Japan and Nikon D-80; Nikon, Tokyo, Japan), and optic nerve configuration with retinal pathologic findings were recorded. Intraocular pressure was measured with a Goldmann applanation tonometer. Visual field testing was performed with frequency doubling technology (FDT; Humphrey Matrix; Carl Zeiss Meditec Inc., Dublin, CA, USA) using the N-30-1 screening protocol. The test location was defined as abnormal if it was not identified after two attempts at a contrast level that identified 99% of the healthy population. If two different test locations were abnormal, a visual field defect was noted in that eye. Frequency doubling technology was administered to participants suspected of having glaucoma and who met any of the following criteria: (1) IOP ≥22 mmHg, (2) horizontal or vertical cup-to-disc ratio (CDR) ≥0.5, (3) nonadherence to the ISNT rule (neuroretinal rim thickness in the following order by quadrant: inferior > superior > nasal > temporal), (4) presence of optic disc haemorrhage (DH), or (5) presence of a retinal nerve fibre layer (RNFL) defect. Frequency doubling technology was repeated if either the rate of fixation errors or the false-positive rate was greater than 0.33, in which case the FDT was determined as invalid for glaucoma classification.

### Definitions of OAG and control groups

The definition of OAG was based on the International Society of Geographical and Epidemiological Ophthalmology criteria and a previous study [18–20]. Patients were defined as having OAG if an open angle was present (PACD >1/4 corneal thickness based on the Van Herick method) and if any one of the following category I or category II diagnostic criteria were met.

Category I criteria were applied to subjects with FDT perimetry results showing a fixation error and false-positive error of one or less. Glaucoma-diagnostic criteria were (1) loss of neuroretinal rim with vertical or horizontal CDR of 0.7 or more or asymmetric CDR of 0.2 or more (both values determined by ≥ 97.5th percentile for the normal KNHANES population), (2) presence of DH, or (3) presence of an RNFL defect. Additionally, the subjects had to show abnormal FDT testing results with at least one location of reduced sensitivity compatible with optic disc appearance or RNFL defect. Criteria II were applied to those with an absence of FDT perimetry results, fixation error, or a false-positive error of two or more with (1) loss of neuroretinal rim and vertical CDR ≥0.9 or asymmetry of vertical CDR ≥0.3 or (2) presence of an RNFL defect compatible with optic disc appearance.

Participants who met the following criteria in both eyes served as controls: (1) IOP ≤21 mmHg, (2) presence of an open angle (PACD >1/4 corneal thickness), (3) non-glaucomatous optic disc (vertical and horizontal CDR <0.7 and inter-eye difference of vertical and horizontal CDR <0.2), (4) absence of DH or RNFL defect, and (5) optic disc not violating the ISNT rule.

After preliminary grading, more detailed grading was performed independently by another group of glaucoma specialists who were masked to the participants’ other information. Any discrepancy between the preliminary and detailed grading was adjudicated by a third group (two glaucoma specialists).

### Statistical analyses

Statistical analysis was performed using STATA version 15.1 (StataCorp, College Station, TX, USA) to account for the complex sampling design. Strata, sampling units, and sampling weights were used to obtain point estimates and standard errors (SEs) of the mean. All data analyses were performed using weighted data, and SEs of the mean of population estimates were calculated using Taylor linearization methods. Participant characteristics were summarised for the entire sample using mean and SE for continuous variables and frequency, percentage, and SE for categorical variables.

Baseline demographic information and clinical parameters were compared between the groups using Pearson’s Chi-square test for categorical variables and general linear models for continuous variables. General linear models were used to examine the relationships between beverages and OAG. For these models, we adjusted for age, sex, BMI, diabetes mellitus, hypertension, total cholesterol levels, heavy drinking, or current smoking. After dividing the participants into five groups according to consumption, we analysed the relationships between consumption and OAG for each beverage. Logistic regression models were used to estimate the odd ratios (ORs) with 95% confidence intervals (CIs). Group 1 (no consumption of beverages) was used as the reference. β-coefficient values and 95% CIs were obtained. To investigate the sex difference between beverage consumption and OAG, we stratified our analyses based on sex and then adjusted for age. ORs and 95% CIs for OAG risk were also obtained. P values were two-tailed, and p < 0.05 was considered statistically significant.

## Results

A total of 6,681 participants (6,358 for normal control, 323 for OAG without treatment) was included in the analysis. The prevalence of OAG was 4.4% (n = 323), including 5.4% (n = 169) among men and 3.5% (n = 154) among women. Patients who met category I diagnostic criteria numbered 276, and those who met category II criteria totaled 47. Among 323 patients with OAG, 310 were newly diagnosed, while 13 were previously diagnosed. Table 1 shows the demographics of study participants. Glaucoma patients more frequently showed the following in relation to normal subjects: men, old age, diabetes, hypertension, and low serum level of high-density lipoprotein cholesterol. Table 2 shows IOP status according to beverage consumption. The IOP status was not significantly different according to consumption of coffee, tea, or soft drinks. Table 3 shows ORs for the presence of OAG according to beverage consumption. After adjusting for relating factors, coffee consumption showed a statistically significant relationship with presence of OAG, while consumption of tea or soft drinks did not show a significant relationship. The OR comparing those who consumed coffee with those who did not consume coffee was 2.06 (95% CI, 1.11–3.82). The association of coffee consumption was significant in men but not in women. Table 4 shows ORs for the presence of OAG according to amount of each beverage consumed. Coffee consumption showed a statistically significant relationship with presence of OAG at all consumption levels but did not show an increased risk of OAG with increased consumption.

**Table 1:** Baseline characteristics of study participants with and without OAG.

	OAG (*n* = 323; 4.4%)		Non-glaucoma (*n* = 6,358; 95.6%)		*p*-value
	Mean or % (SE)	95% CI	Mean or % (SE)	95% CI	
Age, years	49.9 (1.2)	47.6–52.2	41.8 (0.3)	41.2–42.3	<0.001^a^
Men, %	59.9 (3.3)	53.3–66.2	49.0 (0.7)	47.7–50.4	0.002^b^
Current smoker, %	28.7 (3.3)	22.6–35.6	25.8 (0.8)	24.2–27.4	0.363^b^
Heavy drinking, %	60.5 (3.6)	53.4–67.3	60.9 (0.8)	59.3–62.6	0.916^b^
BMI, kg/m^2^	23.7 (0.2)	23.3–24.0	23.7 (0.1)	23.6–23.8	0.954^a^
Waist circumference, cm	81.8 (0.6)	80.6–83.0	80.8 (0.2)	80.4–81.2	0.093^a^
Systolic BP, mmHg	122.4 (1.1)	120.2–124.6	116.4 (0.3)	115.9–117.0	<0.001^a^
Diastolic BP, mmHg	79.2 (0.7)	77.8–80.6	76.4 (0.2)	76.0–76.8	<0.001^a^
Serum glucose, mg/dL	99.5 (1.7)	96.1–102.8	94.8 (0.3)	94.2–95.4	0.007^a^
Total cholesterol, mg/dL	190.0 (2.9)	184.3–195.7	187.4 (0.6)	186.1–188.6	0.393^a^
HDL-C, mg/dL	51.4 (0.9)	49.6–53.2	53.2 (0.2)	52.8–53.6	0.049^a^
LDL-C, mg/dL	114.4 (4.2)	106.1–122.7	112.1 (0.9)	110.3–113.8	0.588^a^
Triglycerides, mg/dL	143.1 (7.0)	129.3–157.0	129.7 (1.8)	126.1–133.2	0.062^a^
Diabetic status					<0.001^b^
DM, %	14.3 (2.4)	10.2–19.7	6.5 (0.4)	5.8–7.3	
Pre-DM, %	17.3 (2.4)	13.0–22.6	15.1 (0.6)	13.9–16.3	
Systemic hypertension					<0.001^b^
Hypertension, %	33.5 (3.4)	27.2–40.4	19.3 (0.6)	18.0–20.6	
Prehypertension, %	25.2 (2.9)	19.8–31.4	22.9 (0.7)	21.4–24.3	
IOP (mmHg)	14.3 (0.2)	13.8–14.7	14.0 (0.1)	13.8–14.1	0.172^a^

BMI, body mass index; BP, blood pressure; CI, confidence interval; DM, diabetes mellitus; HDL-C, high-density lipoprotein cholesterol; IOP, intraocular pressure; LDL-C, low-density lipoprotein cholesterol; OAG, open-angle glaucoma; SE, standard error.

Data are presented as mean (SE) for continuous variables and as percentage (SE) for categorical variables.

^a^General linear model was used for continuous variables.

^b^Chi-square test was used for categorical data.

**Table 2:** The relationship between common beverage intake and intraocular pressure in non-glaucoma subjects.

	Unadjusted β coefficient			Model 1			Model 2		
	Total	Men	Women	Total	Men	Women	Total	Men	Women
	β (95% CI)	β (95% CI)	β (95% CI)	β (95% CI)	β (95% CI)	β (95% CI)	β (95% CI)	β (95% CI)	β (95% CI)
Coffee intake									
None	0	0	0	0	0	0	0	0	0
<6 cups/week	0.23 (-0.08–0.55)	0.63* (0.10–1.16)	-0.06 (-0.41–0.29)	0.22 (-0.10–0.53)	0.63* (0.11–1.16)	-0.05 (-0.40–0.30)	0.14 (-0.19–0.48)	0.57* (0.02–1.12)	-0.14 (-0.50–0.22)
1 cup/day	0.25 (-0.06–0.56)	0.80** (0.30–1.29)	-0.13 (-0.50–0.24)	0.23 (-0.09–0.55)	0.79** (0.29–1.29)	-0.15 (-0.52–0.22)	0.19 (-0.15–0.52)	0.74** (0.24–1.24)	-0.18 (-0.57–0.20)
2 cups/day	0.28 (-0.05–0.61)	0.85** (0.33–1.36)	-0.15 (-0.51–0.22)	0.24 (-0.08–0.57)	0.84** (0.32–1.36)	-0.16 (-0.53–0.20)	0.16 (-0.18–0.49)	0.73** (0.20–1.26)	-0.23 (-0.61–0.16)
≥3 cups/day	0.36* (0.04–0.67)	0.74** (0.25–1.23)	-0.06 (-0.45–0.33)	0.25 (-0.07–0.57)	0.73** (0.24–1.22)	-0.07 (-0.46–0.32)	0.17 (-0.15–0.50)	0.64* (0.15–1.14)	-0.12 (-0.51–0.28)
*p* for trend	0.049	0.031	0.580	0.229	0.037	0.495	0.440	0.088	0.495
*p* for interaction by sex				0.016			0.016		
Soft drinks intake									
None	0	0	0	0	0	0	0	0	0
<6 cups/week	-0.13 (-0.31–0.05)	-0.17 (-0.45–0.12)	-0.21 (-0.42–0.01)	-0.16 (-0.35–0.02)	-0.15 (-0.45–0.16)	-0.17 (-0.40–0.06)	-0.19* (-0.37–-0.01)	-0.10 (-0.40–0.20)	-0.24* (-0.47–0)
1 cup/day	-0.32 (-1.04–0.39)	-0.19 (-1.07–0.70)	-0.97 (-2.09–0.16)	-0.39 (-1.13–0.34)	-0.16 (-1.07–0.76)	-0.90 (-2.04–0.24)	-0.53 (-1.27–0.20)	-0.19 (-1.07–0.69)	-1.12 (-2.39–0.15)
2 cups/day	-0.17 (-1.40–1.06)	-1.06 (-2.39–0.27)	1.13 (-0.63–2.90)	-0.21 (-1.50–1.09)	-1.02 (-2.38–0.35)	1.21 (-0.55–2.97)	-0.12 (-1.42–1.17)	-0.62 (-2.02–0.78)	1.23 (-0.50–2.96)
≥3 cups/day	-0.88 (-4.70–2.95)	-0.84 (-5.01–3.33)	-2.94*** (-3.14–-2.75)	-1.04 (-4.85–2.77)	-0.81 (-4.97–3.35)	-3.04*** (-3.32–-2.77)	-1.15 (-4.7–2.39)	-0.76 (-4.66–3.15)	-3.20*** (-3.62–-2.77)
*p* for trend	0.118	0.185	0.045	0.062	0.259	0.106	0.025	0.359	0.035
*p* for interaction by sex				0.190			0.226		
Tea intake									
None	0	0	0	0	0	0	0	0	0
<6 cups/week	0.12 (-0.05–0.30)	0.18 (-0.1–0.45)	0.04 (-0.17–0.26)	0.13 (-0.05–0.31)	0.20 (-0.08–0.48)	0.07 (-0.14–0.28)	0.16 (-0.03–0.35)	0.21 (-0.09–0.51)	0.11 (-0.11–0.34)
1 cup/day	0.07 (-0.23–0.38)	0.33 (-0.09–0.74)	-0.25 (-0.65–0.16)	0.06 (-0.24–0.36)	0.34 (-0.08–0.75)	-0.24 (-0.64–0.17)	0.06 (-0.25–0.36)	0.26 (-0.17–0.69)	-0.14 (-0.56–0.28)
2 cups/day	0.31 (-0.24–0.86)	0.46 (-0.31–1.23)	0.002 (-0.82–0.83)	0.27 (-0.28–0.83)	0.47 (-0.29–1.24)	0.02 (-0.81–0.85)	0.31 (-0.21–0.84)	0.57 (-0.13–1.27)	-0.07 (-0.93–0.79)
≥3 cups/day	-0.51 (-1.11–0.09)	-0.42 (-1.35–0.51)	-0.63* (-1.26–0)	-0.52 (-1.11–0.07)	-0.42 (-1.35–0.50)	-0.61 (-1.23–0.01)	-0.55* (-1.09–-0.01)	-0.53 (-1.37–0.32)	-0.62* (-1.23–-0.01)
*p* for trend	0.918	0.368	0.182	0.982	0.349	0.228	0.959	0.410	0.329
*p* for interaction by sex				0.353			0.588		

Model 1: adjusted for age and sex.

Model 2: adjusted for age, sex, BMI, DM, systemic hypertension, total cholesterol, alcohol consumption, and smoking.

BMI, body mass index; CI, confidence interval; DM, diabetes mellitus.

Age, BMI, and total cholesterol were adjusted as continuous variables, while sex, DM, systemic hypertension, alcohol consumption, and smoking were adjusted as categorical data. Diabetes mellitus and systemic hypertension were defined as a combination of physician diagnosis and use of blood glucose-lowering or antihypertensive agents. Alcohol consumption was categorized as ‘heavy drinking’ at more than 60 g/day (men) or 40 g/day (women) more than two days per week or ‘other’. Smoking status was classified as ‘current smoker’ or ‘non-smoker’.

*p < 0.05

**p < 0.01, and

***p < 0.001.

**Table 3:** Risk for open-angle glaucoma according to beverage consumptions.

	Number of glaucoma			Unadjusted			Model 1			Model 2		
	Overall	Men	Women	Overall	Men	Women	Overall	Men	Women	Overall	Men	Women
	Case/total	Case/total	Case/total	OR (95% CI)	OR (95% CI)	OR (95% CI)	OR (95% CI)	OR (95% CI)	OR (95% CI)	OR (95% CI)	OR (95% CI)	OR (95% CI)
Coffee intake												
No	23/653	10/196	13/457	1	1	1	1	1	1	1	1	1
Yes	300/6,028	159/2,440	141/3,588	2.11* (1.19–3.74)	3.37** (1.59–7.11)	1.42 (0.66–3.04)	2.05* (1.15–3.65)	3.33** (1.57–7.09)	1.48 (0.67–3.27)	2.06* (1.11–3.82)	3.32** (1.53–7.20)	1.48 (0.61–3.54)
*p* for interaction by sex							0.140			0.237		
Soft drinks intake												
No	143/2,415	67/762	76/1,653	1	1	1	1	1	1	1	1	1
Yes	180/4,266	102/1,874	78/2,392	0.63** (0.45–0.87)	0.57** (0.37–0.86)	0.58* (0.38–0.89)	0.85 (0.61–1.18)	0.84 (0.52–1.35)	0.86 (0.57–1.28)	0.85 (0.61–1.20)	0.87 (0.53–1.42)	0.84 (0.54–1.29)
*p* for interaction by sex							0.901			0.940		
Tea intake												
No	139/2,454	67/922	72/1,532	1	1	1	1	1	1	1	1	1
Yes	184/4,227	102/1,714	82/2,513	0.90 (0.70–1.16)	0.90 (0.62–1.31)	0.84 (0.56–1.28)	1.06 (0.81–1.39)	1.07 (0.73–1.57)	1.05 (0.69–1.59)	1.12 (0.82–1.53)	1.27 (0.83–1.95)	0.997 (0.63–1.58)
*p* for interaction by sex							0.847			0.502		

Model 1: adjusted for age and sex.

Model 2: adjusted for age, sex, BMI, DM, systemic hypertension, total cholesterol, alcohol consumption, and smoking.

BMI, body mass index; CI, confidence interval; DM, diabetes mellitus; OR, odds ratio.

Age, BMI, and total cholesterol were adjusted as continuous variables, while sex, DM, systemic hypertension, alcohol consumption, and smoking were adjusted as categorical data. Diabetes mellitus and systemic hypertension were defined as a combination of physician diagnosis and use of blood glucose-lowering or antihypertensive agents. Alcohol consumption was categorized as ‘heavy drinking’ at more than 60 g/day (men) or 40 g/day (women) more than two days per week or ‘other’. Smoking status was classified as ‘current smoker’ or ‘non-smoker’.

*p < 0.05

**p < 0.01, and

***p < 0.001.

**Table 4:** The relationship between consumption of common beverages and prevalence of open-angle glaucoma.

	Number of glaucoma			Unadjusted			Model 1			Model 2		
	Overall	Men	Women	Overall	Men	Women	Overall	Men	Women	Overall	Men	Women
	Case/total	Case/total	Case/total	OR (95% CI)	OR (95% CI)	OR (95% CI)	OR (95% CI)	OR (95% CI)	OR (95% CI)	OR (95% CI)	OR (95% CI)	OR (95% CI)
Coffee intake												
None	23/653	10/196	13/457	1	1	1	1	1	1	1	1	1
<6 cups/week	72/1,615	33/561	39/1,054	1.88 (1.00–3.56)	3.04* (1.29–7.19)	1.28 (0.55–2.96)	2.07* (1.09–3.91)	3.50** (1.48–8.28)	1.41 (0.60–3.33)	2.09* (1.06–4.13)	3.35** (1.38–8.09)	1.47 (0.58–3.73)
1 cup/day	71/1,571	33/510	38/1,061	1.94* (1.03–3.66)	3.43** (1.50–7.81)	1.25 (0.55–2.88)	1.84 (0.97–3.49)	3.25** (1.41–7.49)	1.21 (0.52–2.82)	1.75 (0.89–3.47)	3.27** (1.37–7.78)	1.08 (0.43–2.72)
2 cups/day	82/1,521	38/581	44/940	2.27** (1.25–4.13)	3.28** (1.45–7.44)	1.79 (0.78–4.10)	2.12* (1.15–3.90)	2.95* (1.29–6.73)	1.86 (0.78–4.41)	2.11* (1.09–4.07)	2.65* (1.12–6.24)	1.97 (0.75–5.20)
≥3 cups/day	75/1,321	55/788	20/533	2.41** (1.30–4.48)	3.66** (1.67–8.03)	1.39 (0.56–3.44)	2.21* (1.18–4.14)	3.56** (1.61–7.87)	1.58 (0.61–4.13)	2.40* (1.22–4.72)	3.98** (1.71–9.25)	1.58 (0.56–4.42)
*p* for trend				0.015	0.093	0.224	0.106	0.265	0.190	0.075	0.171	0.221
*p* for interaction by sex							0.316			0.186		
Soft drinks intake												
None	143/2,415	67/762	76/1,653	1	1	1	1	1	1	1	1	1
<6 cups/week	176/4,167	100/1,816	76/2,351	0.64** (0.46–0.88)	0.58* (0.39–0.89)	0.58* (0.37–0.89)	0.85 (0.61–1.19)	0.85 (0.53–1.36)	0.85 (0.57–1.26)	0.86 (0.61–1.21)	0.88 (0.54–1.44)	0.83 (0.53–1.28)
≥1 cup/day	4/99	2/58	2/41	0.32* (0.11–0.94)	0.14* (0.03–0.63)	0.80 (0.18–3.50)	0.55 (0.18–1.67)	0.27 (0.06–1.31)	1.53 (0.36–6.46)	0.48 (0.13–1.71)	0.12* (0.02–0.98)	1.48 (0.34–6.44)
*p* for trend				0.003	0.002	0.021	0.268	0.309	0.550	0.288	0.360	0.521
*p* for interaction by sex							0.252			0.124		
Tea intake												
None	139/2,454	67/922	72/1,532	1	1	1	1	1	1	1	1	1
<6 cups/week	141/3,423	75/1,340	66/2,083	0.81 (0.62–1.05)	0.79 (0.54–1.16)	0.79 (0.52–1.21)	0.97 (0.73–1.28)	0.95 (0.64–1.41)	1.00 (0.64–1.55)	1.02 (0.74–1.41)	1.13 (0.72–1.77)	0.92 (0.57–1.49)
1 cup/day	25/521	16/234	9/287	1.26 (0.75–2.12)	1.36 (0.66–2.81)	1.00 (0.42–2.38)	1.42 (0.83–2.42)	1.57 (0.75–3.28)	1.17 (0.49–2.77)	1.58 (0.88–2.83)	1.96 (0.89–4.30)	1.19 (0.49–2.89)
2 cups/day	10/148	7/81	3/67	1.57 (0.75–3.29)	1.51 (0.61–3.71)	1.43 (0.36–5.79)	1.83 (0.85–3.92)	1.84 (0.73–4.61)	1.80 (0.43–7.50)	2.10 (0.94–4.69)	2.32 (0.86–6.24)	1.90 (0.45–8.03)
≥3 cups/day	8/135	4/5	4/76	1.18 (0.49–2.85)	1.22 (0.37–3.99)	1.04 (0.31–3.47)	1.23 (0.52–2.91)	1.25 (0.40–3.96)	1.19 (0.34–4.15)	1.08 (0.42–2.78)	0.92 (0.22–3.82)	1.37 (0.39–4.85)
*p* for trend				0.397	0.431	0.984	0.128	0.197	0.504	0.099	0.121	0.485
*p* for interaction by sex							0.987			0.912		

Model 1: adjusted for age and sex.

Model 2: adjusted for age, sex, BMI, DM, systemic hypertension, total cholesterol, alcohol consumption, and smoking.

BMI, body mass index; CI, confidence interval; DM, diabetes mellitus; OR, odds ratio.

Age, BMI, and total cholesterol were adjusted as continuous variables, while sex, DM, systemic hypertension, alcohol consumption, and smoking were adjusted as categorical data. Diabetes mellitus and systemic hypertension were defined as a combination of physician diagnosis and use of blood glucose-lowering or antihypertensive agents. Alcohol consumption was categorized as ‘heavy drinking’ at more than 60 g/day (men) or 40 g/day (women) more than two days per week or ‘other’. Smoking status was classified as ‘current smoker’ or ‘non-smoker’.

*p < 0.05

**p < 0.01, and ***p < 0.001.

## Discussion

Our study indicates that drinking coffee significantly increased risk of OAG in men but not women. Conversely, no significant association was found between consumption of tea or soft drinks and risk of OAG. In addition, coffee consumption was not significantly associated with elevation of IOP.

Many studies have explored the association between caffeinated beverages and IOP or OAG, but there have been conflicting results. The plasma and aqueous levels of homocysteine may be elevated by coffee, which is associated with development of pseudoexfoliation glaucoma and OAG [8,21]. A meta-analysis of randomized controlled trials suggested that coffee consumption raises the serum levels of triglycerides and low-density lipoprotein cholesterol [22]. Another study found that it slightly increased glycosylated haemoglobin (HbA1c) [23]. Higher level of HbA1c and metabolic syndrome were suggested as risk factors for development of glaucoma [24,25]. In addition, coffee contains many ingredients, and it is possible that bioactive components other than caffeine are responsible for glaucomatous optic nerve damage. For example, the acrylamide contained in coffee probably plays a role in neurotoxicity related to conjugation of acrylamide with cysteine residues of presynaptic membrane proteins engaged in neurotransmitter release [26]. As a result, the flow of nerve impulses may be inhibited, coupled with subsequent degeneration of neurons. Oxidative stress is also caused by acrylamide [26].

In this study, there was an inverse association between consumption of soft drinks and IOP mainly in participants who consumed more than 3 cups of soft drinks per day. Excessive intake of soft drinks containing phosphorus additives could cause metabolic acidosis, which might lead to a decrease in IOP [27,28]. However, IOP reduction associated with soft drinks was seen only in women, and sex differences in the impact of soft drinks on IOP remain unknown. This may require further studies.

Caffeine is a methylxanthine derivative and is a component of both tea and coffee. Emerging data have suggested that caffeine-induced vasoconstriction and the subsequent reduction in ocular blood flow may increase the risk of glaucomatous optic neuropathy. Mathew et al reported a significant reduction in cerebral blood flow after ingestion of 250 mg caffeine under double-blind conditions [29]. Vasoconstriction induced by caffeine may result from its inhibitory effect on adenosine, which acts as a potent vasodilator. Some studies presented evidence of increased vascular resistance and decreased blood flow in the optic nerve head and choroidal–retinal circulation after caffeine administration [30,31]. It is possible that altered hemodynamic response may cause ischemic insult and render the optic nerve more sensitive to elevated IOP [32]. Indeed, vascular dysregulation is considered a pivotal factor, especially in the pathogenesis of OAG with low IOP, which is the most common type of glaucoma in Korean people [33]. Patients with OAG may show abnormal vascular responses to caffeine intake; thus, glaucomatous change may occur even with only a tiny alteration in IOP.

Some randomized controlled trials have indicated that ingestion of caffeinated coffee can lead to a significant IOP elevation in participants with or at risk for glaucoma compared with controls taking in equal volumes of fluid [14,34,35]. Kang et al reported via a prospective cohort study that overall regular coffee consumption was not associated with risk of OAG, but subgroup analyses showed a significant adverse correlation between caffeinated coffee and OAG with IOP ≥22 mmHg among those with daily consumption of five or more cups of caffeinated coffee or those with a family history of glaucoma [11]. These authors additionally showed that greater caffeine intake was more adversely related to risk of OAG with elevated IOP in those having a family history of glaucoma. However, Wu et al suggested that coffee consumption was not associated with development of glaucoma [10].

Caffeine is considered to play a role in increasing IOP after drinking coffee [36,37]. Many studies regarding the effect of caffeinated beverages on eyes have reported that caffeine may affect aqueous production and drainage. Although the mechanism is not clearly understood, theoretically, caffeine can raise IOP by inhibiting phosphodiesterase activity, resulting in higher intracellular cyclic AMP level and greater aqueous humour production in the ciliary body [38]. In an animal model of ocular hypertension, dilated intercellular spaces in the nonpigmented ciliary epithelium were observed following intravenous caffeine administration, suggesting caffeine-induced enhancement of aqueous humour transport [39]. Caffeine is also assumed to reduce aqueous humour outflow through the trabecular meshwork by decreasing smooth muscle tone [37]. Although the caffeine effect on aqueous outflow was not seen in healthy individuals, most studies conducted in participants with or at risk of glaucoma have shown a positive association between caffeine intake and IOP [36,37,40,41].

Given that homeostatic regulation of IOP is mainly achieved by aqueous outflow control, IOP may increase significantly in eyes with impaired outflow facility after exposure to provocative factors such as caffeine or fluid intake. The Blue Mountains Eye Study, a population-based, cross-sectional study, demonstrated the significant effect of coffee consumption on IOP elevation, especially in participants with OAG [42]. Li et al reported that caffeine had little effect on IOP in normal individuals, while patients with ocular hypertension or glaucoma showed significant IOP elevation [7]. Glaucoma patients show higher resistance to aqueous outflow in comparison with people of similar ages without glaucoma, and this finding may further explain the mechanism of IOP elevation in eyes with OAG after coffee consumption [43]. In our study, there was no significant difference in IOP according to coffee consumption between normal participants and glaucoma patients. Given that most study patients had OAG without high IOP, which indicates relatively normal outflow facilities, our study supports lack of influence on IOP by caffeine.

Dietary intake of phytochemicals and flavonoids in tea has been observed to have antioxidant and neuroprotective effects associated with health benefits [44]. Wu et al reported that individuals who drink hot tea had a lower risk of developing glaucoma [10]. Based on self-reported questionnaires, participants drinking at least one cup of hot tea daily showed a lower risk of glaucoma compared with those not drinking hot tea, whereas consumption of caffeinated coffee or soft drinks was not significantly associated with overall glaucoma risk. Tea contains less caffeine than coffee but more flavonoids and phytochemicals, which have been suggested to play a protective role in development or progression of glaucoma [45–47]. However, the effect of tea consumption on glaucoma remains unclear. In our study, which was performed with a larger group of participants from a single ethnic population, the results support a positive association between coffee consumption and risk of OAG. Conversely, regarding the effect of tea or soft drinks on OAG, we could not find any significant association. Differences in study methodology and ethnicity in study participants might account for this discrepancy.

In our study, the adverse association between coffee consumption and OAG was observed particularly in men, whereas this association was not significant in women. We cannot explain why this is, though men and women have different body structures and serum hormone levels. Some studies have reported difference in prevalence and risk factors of OAG between men and women [4–6,48]. One study reported that serum glutamate concentration was significantly higher in men than in women, possibly due to the effects of estrogen and progesterone [49]. The tissue responses of men and women for glaucomatous insult seem fundamentally different. Estrogen-related effects such as IOP reduction or neuroprotection have been suggested as possible mechanisms to explain the sex difference [50,51]. Regarding coffee consumption and OAG, Kang et al. showed that increasing intake of caffeine was significantly related to higher risk of OAG in women, not in men, but this association was only statistically significant in a group of women with high IOP (≥22 mmHg) [11]. Conflicting results from our study may be due to differences in study population (cohort-based vs. population-based) and methodology (incident vs. prevalent OAG). Overall, there have been controversies about sex predilection in OAG, and the mechanism of the sex-specific association remains unknown. Further studies are warranted to disclose the underlying pathophysiology.

Our study had some limitations. Because this study was an observational and cross-sectional design, the incidence of OAG and the causality between beverage consumption and OAG could not be determined. We could not analyze the types of tea consumed or the drink methods of beverage due to lack of data on aspects including beverage size. Since caffeinated beverage is not the same as caffeine, the association of caffeinated beverage with OAG should not be equated with that of caffeine. Considering the nature of a questionnaire, because the survey used depends on recall, the information obtained was likely not completely accurate. Furthermore, the role of family history, which is a strong risk factor for OAG, could not be evaluated in the association between coffee consumption and risk of OAG. However, there was a clear distinction between people who do not drink coffee and those who drink it, and our results showed that coffee has a detrimental relationship with OAG in Koreans. Unmeasured or residual confounding factors may contribute to unexpected analytical bias. In addition, the visual field was examined by FDT rather than by Humphrey field analysis, which is the test of choice for visual field testing. However, FDT is a fast, reliable, large-scale screening method that can detect glaucomatous visual field defects earlier than standard automated perimetry [52,53]. Angle status was assessed using Van Herick methods, not gonioscopic examination. Although this study has limitations, the strengths of our study include its representation of a South Korean population and its relatively large sample size and high response rate.

Additional consideration should be given to the fact that epidemiologic studies investigating the effects of caffeine on glaucoma are complicated due to the difficulty in estimating dietary caffeine intake, great individual variability in caffeine sensitivity, and poor understanding of pathological processes in the eye [54,55]. Furthermore, ethnic differences in the prevalence of glaucoma as well as in physiological response to caffeine have been reported consistently, which suggest the need for research on the relationship between caffeine intake and OAG in different ethnicities [56–58].

The main stressor for glaucomatous damage is relatively higher IOP than that tolerable for the optic nerve. The threshold of response to stress is different depending on age, sex, ethnicity, and other factors. In this population-based study with data from KNHANES, we identified a significant association between coffee consumption and risk of OAG, particularly in men, while consumption of tea or soft drinks was not significantly associated with OAG. According to these results, a limitation on drinking coffee may be helpful for decreasing the risk of OAG. Further studies are required to find the mechanisms and determine the sex differences in caffeine effects on OAG. If further studies are carried out and good results are revealed, precise advice to the patient will be available.

## Supporting information

S1 Table Baseline characteristics of study participants according to coffee consumption. (PDF)

S2 Table Baseline characteristics of study participants according to categories of coffee consumption. (PDF)
