# Supplementary material for: Effects of consumption of coffee, tea, or soft drinks on open-angle glaucoma: Korea National Health and Nutrition Examination Survey 2010 to 2011
Source: PLoS One. 2020 Jul 20;15(7):e0236152. doi: 10.1371/journal.pone.0236152 (PMC7371211; doi:10.1371/journal.pone.0236152)
Supplement: S1 Table — (PDF) [file pone.0236152.s001.pdf]

S1 Table. Baseline characteristics of study participants according to coffee consumption.

	Coffee consumption		*p*-value
	Yes (n = 6,028; 90.6%)	No (n = 653; 9.4%)	
Age, years	42.3 (0.3)	40.8 (0.8)	0.058
Men, %	50.5 (0.7)	39.7 (2.5)	<0.001
Current smoker, %	27.2 (0.9)	13.7 (2.2)	<0.001
Heavy drinking, %	62.6 (0.9)	44.8 (2.5)	<0.001
BMI, kg/m^2	23.7 (0.1)	23.1 (0.2)	0.001
Waist circumference, cm	81.0 (0.2)	79.1 (0.5)	<0.001
Systolic BP, mmHg	116.8 (0.3)	115.4 (0.8)	0.071
Diastolic BP, mmHg	76.7 (0.2)	74.8 (0.5)	0.001
Serum glucose, mg/dL	95.1 (0.3)	93.9 (1.0)	0.222
Total cholesterol, mg/dL	187.8 (0.6)	184.0 (2.1)	0.078
HDL-C, mg/dL	53.1 (0.2)	53.6 (0.7)	0.405
LDL-C, mg/dL	112.6 (0.9)	108.3 (2.8)	0.151
Triglycerides, mg/dL	130.1 (1.7)	131.5 (9.1)	0.885
Diabetic status			0.112
DM, %	6.8 (0.4)	7.4 (1.2)	
Pre-DM, %	15.5 (0.6)	11.8 (1.6)	
Systemic hypertension			0.466
Hypertension, %	20.1 (0.7)	18.2 (1.6)	
Prehypertension, %	23.1 (0.8)	22.0 (2.3)	
IOP (mmHg)	14.0 (0.1)	13.7 (0.1)	0.053

BMI, body mass index; BP, blood pressure; CI, confidence interval; DM, diabetes mellitus; HDL-C, high-density lipoprotein cholesterol; IOP, intraocular pressure; LDL-C, low-density lipoprotein cholesterol; SE, standard error.

Data are presented as mean (SE) for continuous variables and as percentage (SE) for categorical variables.
