# Supplementary material for: Effects of consumption of coffee, tea, or soft drinks on open-angle glaucoma: Korea National Health and Nutrition Examination Survey 2010 to 2011
Source: PLoS One. 2020 Jul 20;15(7):e0236152. doi: 10.1371/journal.pone.0236152 (PMC7371211; doi:10.1371/journal.pone.0236152)
Supplement: S2 Table — (PDF) [file pone.0236152.s002.pdf]

S2 Table. Baseline characteristics of study participants according to categories of coffee consumption.

	None (n = 653; 9.4%)	<6 cups/week (n = 1,615; 25.8%)	1 cup/day (n = 1,571; 22.2%)	2 cups/day (n = 1,521; 21.2%)	≥3 cups/day (n = 1,321; 21.5%)	p for trend
Age, years	40.8 (0.8)	38.6 (0.5)	43.5 (0.5)	44.4 (0.4)	43.3 (0.4)	<0.001
Men, %	39.7 (2.5)	47.6 (1.6)	42.4 (1.5)	45.8 (1.5)	67.1 (1.4)	<0.001
Current smoker, %	13.7 (2.2)	20.2 (1.4)	20.3 (1.4)	22.1 (1.5)	47.6 (1.6)	<0.001
Heavy drinking, %	44.8 (2.5)	60.0 (1.5)	56.0 (1.7)	64.3 (1.7)	70.7 (1.5)	<0.001
BMI, kg/m^2	23.1 (0.2)	23.4 (0.1)	23.7 (0.1)	23.9 (0.1)	24.0 (0.1)	<0.001
Waist circumference, cm	79.1 (0.5)	79.7 (0.3)	80.8 (0.3)	81.5 (0.3)	82.5 (0.4)	<0.001
Systolic BP, mmHg	115.4 (0.8)	115.5 (0.5)	117.1 (0.5)	117.3 (0.5)	117.7 (0.5)	<0.001
Diastolic BP, mmHg	74.8 (0.5)	75.3 (0.4)	76.5 (0.3)	77.0 (0.3)	78.2 (0.4)	<0.001
Serum glucose, mg/dL	93.9 (1.0)	94.3 (0.6)	94.7 (0.6)	95.9 (0.6)	95.7 (0.7)	0.032
Total cholesterol, mg/dL	184.0 (2.1)	182.4 (1.1)	187.4 (1.3)	190.7 (1.1)	192.0 (1.1)	<0.001
HDL-C, mg/dL	53.6 (0.7)	54.0 (0.4)	53.2 (0.4)	53.0 (0.4)	51.9 (0.4)	<0.001
LDL-C, mg/dL	108.3 (2.8)	107.3 (1.6)	112.4 (1.8)	114.4 (1.7)	117.6 (1.7)	<0.001
Triglycerides, mg/dL	131.5 (9.1)	119.2 (3.0)	130.3 (3.3)	131.0 (3.1)	141.9 (3.7)	0.003
Diabetic status						0.111
DM, %	7.4 (1.2)	7.2 (0.8)	7.2 (0.8)	7.3 (0.8)	5.3 (0.7)	
Pre-DM, %	11.8 (1.6)	13.6 (1.0)	13.9 (1.1)	18.2 (1.3)	16.8 (1.2)	
Systemic hypertension						0.086
Hypertension, %	18.2 (1.6)	18.3 (1.2)	21.1 (1.2)	20.6 (1.3)	20.7 (1.3)	
Prehypertension, %	22.0 (2.3)	19.8 (1.2)	24.1 (1.4)	22.6 (1.3)	26.3 (1.5)	
IOP (mmHg)	13.7 (0.1)	14.0 (0.1)	14.0 (0.1)	14.0 (0.1)	14.1 (0.1)	0.050

BMI, body mass index; BP, blood pressure; CI, confidence interval; DM, diabetes mellitus; HDL-C, high-density lipoprotein cholesterol; IOP, intraocular pressure; LDL-C,

low-density lipoprotein cholesterol; SE, standard error.

Data are presented as mean (SE) for continuous variables and as percentage (SE) for categorical variables.
